# Rabies in Military Personnel: Epidemiology, Clinical Challenges, and Strategies for Prevention and Management

**DOI:** 10.3390/tropicalmed11090243

**Published:** 2026-08-26

**Authors:** Elodie Monchatre-Leroy, Diana Isabela Costescu Strachinaru, Oula Itani, Sophie Wensierski, Alexandre Servat, Patrick Soentjens

**Affiliations:** 1 Laboratoire Anses de la Rage et de la Faune Sauvage de Nancy, 54220 Malzéville, France; elodie.monchatre-leroy@anses.fr (E.M.-L.); alexandre.servat@anses.fr (A.S.); 2 Unité IDEALISS, Institut Polytechnique UniLaSalle, Université d’Artois, 76134 Mont-Saint-Aignan, France; 3 Centre for Infectious Diseases, Queen Astrid Military Hospital, 1120 Brussels, Belgium; psoentjens@itg.be; 4 Infectious Diseases, CHIREC Hospitals Group, 1160 Brussels, Belgium; 5 Institut Pasteur, Centre Médical, Centre d’infectiologie Necker-Pasteur, 75015 Paris, France; oula.itani@pasteur.fr; 6 24th Veterinary Group, 3rd Army Medical Center, 51600 Suippes, France; sophie.wensierski@intradef.gouv.fr; 7 Department of Clinical Sciences, Institute of Tropical Medicine, 2000 Antwerp, Belgium

**Keywords:** rabies, lyssavirus, rabies pre-exposure prophylaxis (PrEP), rabies post-exposure prophylaxis (PEP), military, deployment, animal bites, neglected disease

## Abstract

For deployed military personnel, a single exposure from a rabies-infected animal can become a death sentence. With a case fatality rate approaching 100%, rabies remains a serious, yet insufficiently acknowledged hazard for service members, for whom prevention in operational environments poses unique challenges. We conducted a systematic literature search to identify relevant studies on rabies risk in armed forces personnel. By analysing rabies-related fatalities in the military, this review highlights the operational challenges posed by animal bites during deployment, including underreporting and delayed access to care, which compromise timely post-exposure prophylaxis (PEP) administration. Special emphasis is placed on prevention strategies, including pre-exposure prophylaxis (PrEP) and animal control protocols tailored to military settings. Its aim is to provide a comprehensive, evidence-based resource for healthcare professionals involved in the protection of deployed forces against this ancient yet still deadly infectious disease.

## 1. Introduction

Rabies is a viral zoonosis present on all continents, except Antarctica, and is a serious public health problem in over 150 countries and territories, mainly in Asia and Africa [1]. Although any mammal, both wild and domestic, is capable of contracting rabies, dogs are responsible for almost 99% of human rabies cases [1]. Human rabies is considered the highest case-fatality ratio infectious disease, with almost 100% of cases resulting in the death of the infected individual [2,3]. In the rare instances of survival, most patients remain with severe neurological sequelae [4]. Rabies claims an estimated 59,000 lives per year worldwide, despite being vaccine-preventable [1,5]. Poor rural populations living in endemic countries, particularly children under 15, bear the brunt of the disease and account for 40% of the deaths [1]. The risk among international travellers is relatively low, with a meta-analysis estimating an incidence rate of potential travel-related rabies exposures at 4 per 1000 person-months [6].

Military personnel should be considered a high-risk population for rabies exposure, given that animal-bite injuries are common and that they can be deployed to enzootic regions [7,8,9,10,11]. Military duties often involve a wide range of outdoor activities, increasing the risk of contact with potentially ill stray dogs or other animals. For certain military occupations, such as veterinary staff, working dog handlers, workers who have animal control duties, laboratory employees who work with rabies-suspect samples, and special operations individuals, the risk is even higher [8]. Moreover, armed conflicts often lead to the collapse of healthcare and veterinary infrastructures, as well as shortages of trained workforce. These disruptions significantly hinder infection prevention efforts and undermine public health initiatives, including vaccination campaigns and rabies surveillance for animals, increasing the human risk. Using open-source epidemic intelligence to gain information about unfolding epidemics, Kannan et al. [12] detected more than twice as many human rabies reports after the beginning of the Ukrainian conflict compared with the previous six months, attributed to an increased number of displaced domestic dogs.

The recent death of a 44-year-old man from Iași County, Romania, in mid-July 2025, following a stray dog bite in February 2025, marked the first confirmed autochthonous human case of terrestrial rabies in Romania and the European Union/European Economic Area since 2012 [13]. This case served as a stark reminder that rabies is a global concern for all military forces particularly in border regions where control measures may be weak, and cross-border movement of unvaccinated animals is more likely.

This review aimed to serve as a practical guide to rabies management in military settings by, first, summarizing key aspects of rabies and, second, examining rabies-specific considerations in the armed forces through selected publications, including reported fatalities among military personnel and various strategies for the prevention and management of rabies.

## 2. Rabies Overview

### 2.1. Pathogen

**Figure 1:**
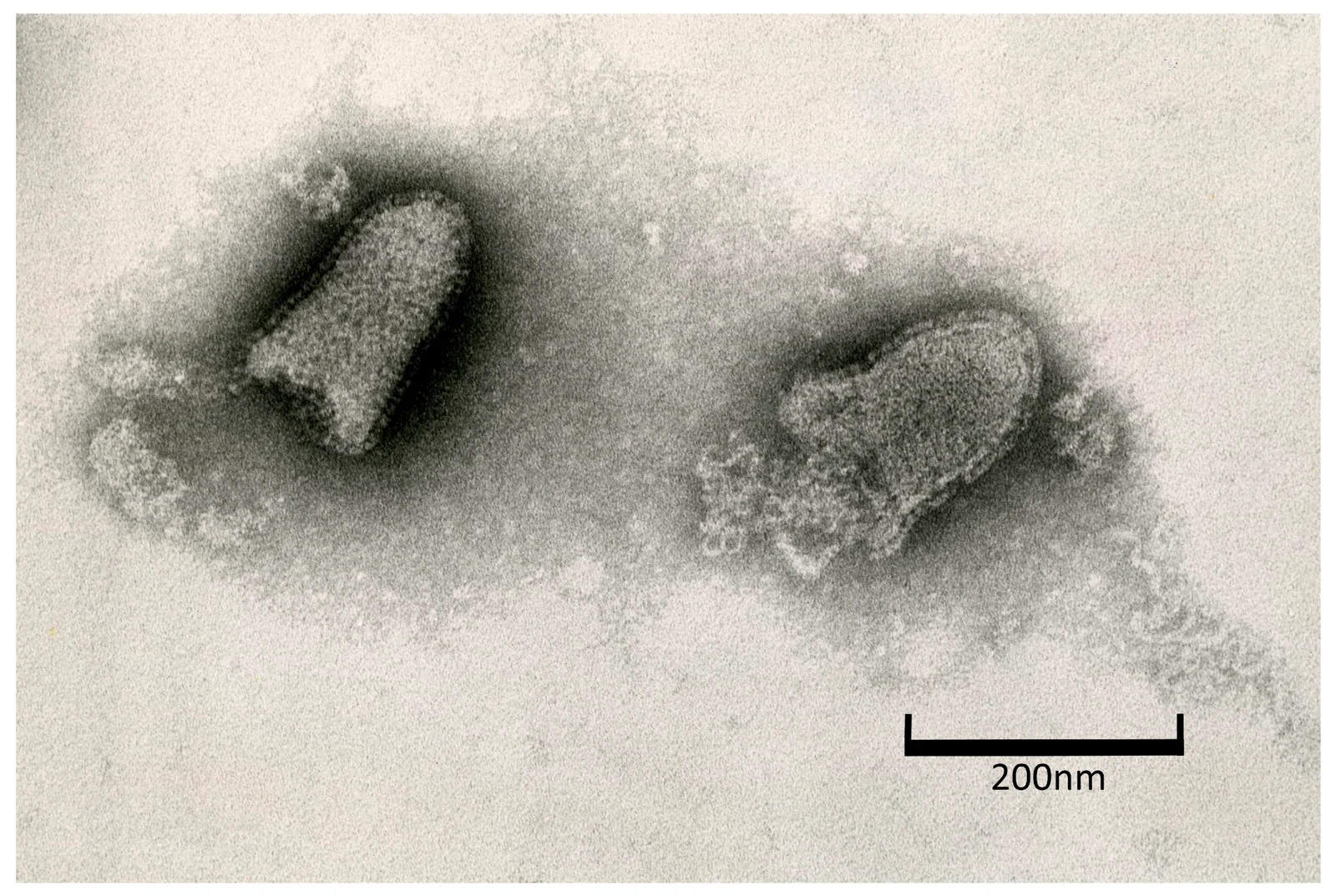
Transmission electron micrograph showing two rabies virions with their distinctive bullet shape. Credit: Institute Pasteur/Monique Lafon and Charles Dauguet.

Rabies virus, *lyssavirus rabies* (RABV), the causative agent of rabies, is a ribonucleic acid (RNA) virus of the genus *Lyssavirus* in the family *Rhabdoviridae* (Figure 1).

The virus consists of a complex of genomic RNA and nucleoprotein, contained within a lipid envelope derived from the host cell membrane [14]. The envelope glycoprotein is the target antigen for antibodies produced following vaccination. Vaccines are produced from RABV, and cross-immunity against other lyssaviruses decreases as phylogenetic distance increases.

RABV is highly resistant to cold temperatures, remaining viable and infectious for prolonged periods (years) when stored at −70 °C, and surviving for up to 6 days at 5 °C. It can also persist during putrefaction, remaining infectious in decomposing carcasses for up to 18 days depending on environmental conditions. In contrast, RABV is rapidly inactivated by desiccation, such as exposure to sunlight for 1.5 h at 30 °C, and by commonly used disinfectants, including iodine, 70% alcohol, and 2% chlorhexidine gluconate solutions [15,16,17].

RABV is the most common member of the Lyssavirus genus, with a large distribution and long history of study. The genus currently includes 19 species recognized by the International Committee on Taxonomy of Viruses [17]. The other lyssaviruses, whose reservoirs (when identified) are various bat species, can also cause fatal rabies-like disease (clinically indistinguishable from rabies) in mammals and humans [18].

### 2.2. Transmission Route

The most frequent transmission mode of rabies in humans is by bite of a rabid animal or the contamination of scratch wounds by virus-laden saliva [1,6]. Other rare transmission routes exist, such as improper manipulation of the body or brain of animals suspected of having rabies, contact between infected saliva with mucosa, or grafting of an infected tissue [1,6]. Aerosol exposure such as in caves inhabited by large colonies of bats or due to a centrifugation incident has been postulated but not scientifically demonstrated [19]. Lyssaviruses do not survive for prolonged periods on dry surfaces; consequently, transmission through contaminated surfaces or fomites is not considered a significant route of infection.

### 2.3. Epidemiology

Lyssaviruses are found on every continent except Antarctica [1]. All mammals are susceptible to rabies virus infection and, in theory, capable of transmitting the virus; however, only a small subset, known as rabies maintenance hosts, are capable of actually spreading the disease [20]. RABV reservoirs are carnivorous mammals and bats. Transmission within these reservoir hosts is facilitated by the aggressive behaviour of rabid carnivores and the blood-feeding behaviour of hematophagous bats (subfamily Desmodontinae) in South America [21]. Rabies epidemiology varies geographically. Because the period of virus shedding is poorly understood in all mammalian species except dogs, cats, and ferrets, any exposure from another mammal should be assessed on a case-by-case basis for rabies risk [22].

Estimates of the global burden of rabies are likely to underestimate the true disease burden because surveillance systems for human and animal cases remain inadequate in many countries. Globally, the burden of rabies is distributed primarily throughout Africa and Asia, where the domestic dog remains the most significant source of human exposure [1,5]. Approximately 39% of the estimated global cases of human rabies occur in Africa [22]. While dogs remain the primary source of human exposures, rabies viruses also circulate enzootically and independently among several other terrestrial carnivores, such as black-backed jackals (*Canis mesomelas*) and yellow mongooses (*Cynictis penicillata*). Moreover, in Africa, several bat species maintain other lyssaviruses [22]. Approximately 60% of the estimated global cases of human rabies occur throughout Asia and the Middle East. As in Africa, most of these cases are associated with uncontrolled rabies in the dog population. India bears the highest burden of human rabies deaths worldwide, accounting for approximately 36% of cases [22,23].

While rabies is still present in Europe, its incidence is low [23]. This is largely attributable to the successful elimination of canine rabies across Western and Central Europe in the early 20th century, as well as to the effective eradication of fox-mediated rabies, the primary reservoir in Western Europe, through widespread oral vaccination campaigns. Nevertheless, foci of fox rabies persist in Central and Eastern European countries. Moreover, raccoon dogs, an invasive species (*Nyctereutes procyonoides)*, have become the second most affected species [24]. The importation of rabid dogs into rabies-free areas also continues to pose risk, as seen in France, Spain, and the Netherlands [25].

### 2.4. Pathogenesis

After the infecting exposure, the virus spreads to neurons through the neuromuscular junction. RABV enters primary motor neurons, after which it undergoes retrograde axonal transport to the neuronal cell body, where replication and assembly occur, followed by transport to another synaptic terminal, where viral budding initiates subsequent cycles of infection and neuron-to-neuron transmission. This slow process of trans-synaptic spread continues until RABV is widely distributed in the CNS [18].

Once in the brain, virus replication drives a series of systemic disease signs indicative of neurological involvement [26]. From a pathogenic perspective, RABV is one of the few microorganisms with a near 100% fatality rate following the onset of clinical signs [1]. The virus causes an acute meningoencephalitis with death thought to result from massive viral replication in the brain leading to cardiac and/or multisystem failure [27]. Despite much research into RABV biology, the mechanism by which RABV infection causes fatal disease is still not fully elucidated [27]. Symptomatic rabies patients die with only mild histological lesions in the brain and without significant loss of neurons [27]. In the late stages of infection, the spread of RABV occurs by centrifugal propagation of RABV to diverse end organs, such as skin, hair follicles, salivary glands and muscle fibres [27]. To reach the salivary gland from the CNS, RABV travels anterogradely through autonomic ganglia, reversing the retrograde neuronal transport that occurs early in infection [28,29]. For much of the course of the disease, RABV infection remains clinically asymptomatic and is characterized by a prolonged incubation period. Clinical signs of neurological dysfunction are present only at late stages of the disease, when the virus reaches the CNS [1,3,28]. Rabies then rapidly deteriorates the health of the host and leads to death from respiratory or heart failure, usually within days [27]. The rate of progression of rabies infection seems to be determined in part by viral factors that differ among variants, and possibly among lyssavirus species. The inoculum also influences disease progression, with higher viral loads resulting in shorter incubation times and, consequently, more rapid deterioration of the host [3,28]. Conversely, lower inocula appear to lengthen the incubation time and possibly increase chances of an abortive infection, where the virus enters the host but cannot replicate sufficiently to invade the CNS [28]. Finally, the severity and location of the wound also influence incubation. Exposures on the head, face or neck are often associated with shorter incubation periods because of the proximity of these sites to the CNS. Moreover, the face and hands are highly innervated tissues, providing the virus with direct access to peripheral nerves [1,28].

### 2.5. Symptoms and Clinical Manifestations

Clinical manifestations of rabies are extremely varied, depending on the encephalic zone where the virus multiplies. However, despite their variations, they share common features, regardless of mammalian species (including humans). The clinical progression of rabies is traditionally categorized into three phases with a maximum total duration of 15 days: prodromal, acute excitative (furious), and paralytic (dumb or end-stage) [30]. This classification, however, has limited diagnostic or prognostic value in practice due to the considerable variability in clinical presentation and the inconsistent duration of each phase. The prodromal phase usually occurs two weeks to three months after exposure, although considerably shorter or longer incubation periods were reported. Lasting approximately 1 to 3 days, it is characterized by subtle, nonspecific behavioural or neurologic changes (loss of appetite, prostration, locomotor disturbances, frequent and unusual vocal sounds) that tend to escalate quickly [30]. The furious phase follows with excessive salivation, hypersensitivity (characterized by exaggerated responses to external stimuli), and aggressiveness (which may be absent). The third phase is marked by the onset of paralysis, succeeded by clinical deterioration that is typically rapid, with death occurring within a few days. In some cases, animals may die suddenly with little to no preceding clinical signs [30]. In addition to these species-independent clinical features, each species can manifest more specific traits [28]. In humans, additional symptoms were reported during the furious phase, such as a tingling sensation at the site of exposure, paranoia, terror, and hallucinations. The most classical sign, hydrophobia, develops in approximately 50–80% of cases [31]. This may begin with throat discomfort or difficulty swallowing, while attempts to drink provoke painful spasms of the diaphragm and other inspiratory muscles lasting 5–15 s. Over time, even the sight, sound, or mention of water can trigger these spasms. A similar phenomenon, aerophobia, is characterized by spasms triggered by air currents coming into contact with the skin [31]. In 20–30% of cases, human rabies may also progress in two phases: prodromal and paralytic. This acute flaccid paralysis form of rabies has a longer clinical course, and is often misdiagnosed, contributing to the under-reporting of the disease [32]. Cardiac manifestations such as arrhythmias including wandering atrial or nodal pacemaker activity, sinus tachycardia or bradycardia, supraventricular or ventricular ectopy, and more severe conditions such as heart failure or cardiac arrest may occur in both forms. Ultimately, regardless of the clinical manifestations, rabies-related death results from progressive paralysis, coma, and asystole [5].

Bats, which are the animal reservoir species for all lyssaviruses, are the only mammals in which infection is not always fatal [27,33,34,35,36]. Rabid bats often lose their ability to fly or do not fly well and they rarely become aggressive. Bats that are active during the day, exhibit unusual behaviour, crawl on the ground, or are found in locations where bats are not typically present may be rabid. Such bats are often the most easily approached. According to George et al., exposure to rabies virus in bats may result in two outcomes: either a non-lethal or a fatal form of the disease [37].

### 2.6. Biological Features and Diagnosis Confirmation

Diagnosis of rabies based solely on clinical features, especially in the absence of a history of exposure, is challenging and unreliable, therefore laboratory confirmation should be performed whenever possible [23,38]. Specific laboratory tests are required to confirm rabies and may be performed on clinical specimens obtained antemortem or postmortem in humans, but only postmortem in animals. Various laboratory techniques are available for the diagnosis of rabies, including detection of viral antigens, virus-specific antibodies, viral nucleic acid, and virus isolation (Table 1).

Regardless of the diagnostic method, analysis performed on postmortem brain tissue samples usually yield better sensitivity and specificity with a higher negative predictive value. The collection of the postmortem samples, however, can be challenging for biosecurity and technical reasons [39]. Conversely, antemortem samples can be easier to collect, but harder to interpret due to lower specificity and sensitivity [39]. Antemortem analyses are only used in humans. Indeed, early antemortem diagnosis is crucial for clinicians, as it guides the decision between palliative care for rabies cases and therapeutic options for non-rabies cases.

In the early stages of human illness, the virus can very occasionally be isolated from saliva or CSF [31]. Recently, Embregts et al. conducted a prospective study in Karachi, Pakistan, which demonstrated that RABV ribonucleic acid (RNA) can be detected from wound swabs collected from unwashed dog-bite wounds within 24 h of exposure [41]. If validated, this approach could offer possible perspectives to assess human exposure in endemic setting.

Serological tests are generally not useful for detecting rabies, as detectable antibody production occurs later in disease progression or do not occurs at all [39]. They are mainly used to measure post-vaccination antibody levels in humans and animals, with rabies virus neutralizing antibody (RVNA) titres ≥ 0.5 IU/mL indicating adequate seroconversion [5,39].

The availability of adequate laboratory capacity, however, greatly impacts rabies surveillance and elimination efforts worldwide, as access to confirmatory testing remains extremely limited in many endemic countries [38].

### 2.7. Pre-Exposure Prophylaxis (PrEP) and Post-Exposure Prophylaxis (PEP)

The main purpose of pre-exposure vaccination is to provide lifelong immunological priming, ensuring a specific adaptive immune response to rabies for humans and animals. Various rabies vaccines, such as purified chicken embryo cell (PCECV) and human diploid cell (HDCV) vaccines, are marketed depending on the country and may be interchangeable [5]. In 2018, the WHO revised its recommendations for PrEP, which now consists of a 2-dose regimen (days 0 and 3) with either a 2-site ID or a 1-site IM vaccine administration [5,39].

In case of potential rabies exposure, PEP should be started as soon as possible, especially if there is a strong suspicion of rabies in the animal responsible for human exposure [1,5]. PEP is possible because of the very long incubation period of rabies, which makes the vaccine effective even when administered after potential exposure. The two essential components of PEP are local treatment of wounds and immunization, including the administration of both vaccine and rabies immunoglobulin (RIG) when needed [1,5]. The importance of immediate and thorough washing of all wounds with soap and water for 15 min cannot be overemphasized, since it may be one of the most effective measures for preventing rabies by reducing the risk of contamination by 90%. The antiseptic of choice is iodine or chlorhexidine [1,15].

After a potential exposure, the risk for rabies and the need for PEP should always be assessed by a physician [26]. The decision to initiate PEP should consider the probability that the animal is infected (depending on epidemiological risk factors) and the patient’s risk category based on the WHO exposure criteria (animal, region, type of contact and immune status) [5,39]. PEP is most effective when administered promptly, ideally within the first 24 h after exposure [5,39]. The rabies PEP regimen in individuals that have received previous PrEP consists of a two-dose intramuscular (IM) or intradermal (ID) regimen of rabies vaccine (on days 0 and 3) or a four-site ID regimen on day 0. Rabies PEP vaccination in naïve individuals consists of 4 IM injections of rabies vaccine over a period of 21 to 28 days—Zagreb regimen: day 0 (two doses), 7 and 21 or modified Essen regimen: day 0, 3, 7, 14 to 28—or two-site ID vaccination on days 0, 3 and 7 [5,39]. Human or equine rabies immunoglobulin (HRIG or ERIG) should be administered as soon as possible after category III exposures in individuals who have not received previous PrEP, and in immunocopromised individuals, regardless of the category or previous PrEP (Table 2) [5,39]. If not initially available, RIG can be given up to 7 days after the first rabies vaccine. After this period, circulating virus-neutralising antibodies (VNAs) begin to appear and RIG is no longer recommended [5,39].

## 3. Rabies in the Armed Forces

### 3.1. Methods

We conducted a systematic literature search to identify articles on rabies and animal bites in military personnel. Two separate searches were performed using the PubMed database (https://pubmed.ncbi.nlm.nih.gov/, accessed on 24 February 2026), without language or date restrictions. The searches were conducted until 24 February 2026. The first search string was (“military” OR “armed forces” OR “military personnel” OR “soldier” OR “army”) AND “rabies”. The second was (“military” OR “armed forces” OR “military personnel” OR “soldier” OR “army”) AND “animal bites” OR “dog bites”. Table 3 presents the inclusion and exclusion criteria.

### 3.2. Literature Search Results

We identified 285 articles corresponding to the search with the words “military” OR “armed forces” OR “military personnel” OR “soldier” OR “army” AND “rabies”. After screening titles and abstracts, when available, 37 articles were considered potentially relevant. Of these, one full-text article could not be retrieved, leaving 36 articles for analysis [8,10,11,12,26,42,43,44,45,46,47,48,49,50,51,52,53,54,55,56,57,58,59,60,61,62,63,64,65,66,67,68,69,70,71,72]. Reference list screening identified two additional relevant articles [7,73]. The search strategy using the terms “military” OR “armed forces” OR “military personnel” OR “soldier” AND “animal bites” OR “dog bites”, yielded 566 articles. After duplicate records were removed, the remaining 506 articles were screened, resulting in the selection of four relevant articles [9,74,75,76].

Most articles concerned the US military (22/42, 52%), troops deployed overseas (19/42, 45%), and epidemiological data on PEP (14/42, 33%) (Table 4). Publication dates ranged from 1962 to 2026. Depending on the topic covered, the period addressed in an article may be highly specific, as in reports of clinical rabies cases, or may span a much longer timeframe, as in epidemiological studies of PEP. The longest period was 9 years (2001 to 2010) [7].

The topics covered focused on military-specific contexts, including exposure to rabies transmission risks that may differ from those encountered in their home countries, as well as force protection through medical measures (PEP and vaccination) and control of the animal reservoir.

### 3.3. Reported Rabies Fatalities in the Military

We identified a total of six cases of rabies death described in the 20th and 21st centuries among soldiers. All cases were acquired during international deployment (Table 5).

The first case was a 27-year-old Dutch soldier that died of rabies in the Utrecht military hospital in 1950, seven months after being deployed to Indonesia. The infecting moment and animal were not identified. During his hospital stay, he bit two staff members, which were subsequently vaccinated against rabies, without further incident [54].

The second case involved a US serviceman who was bitten by a dog on the finger while serving in Vietnam. Despite being started on rabies antiserum and duck embryo vaccine approximately 36 h later, the patient died 108 days after the bite [44]. The authors concluded that the failure of PEP was probably due to the serum being administered too late. The third case was described in a US service member deployed to Vietnam and bitten by a stray puppy [55].

The fourth case was a 23-year-old female US Peace Corps (PC) volunteer working in Kenya in 1983, where she was bitten by an 8-week old puppy (adopted locally), approximatively 6 months after completing a 3-dose ID PrEP series with Human Diploid Cell Vaccine (HDCV) [56,76]. She developed symptoms of rabies 69 days after the bite and, at that time, no rabies VNAs were detected in her serum [76]. A survey in other PC volunteers who had received ID PrEP with the same lot of vaccine showed that 82% had no detectable VNA titers [76,77]. An investigation of the possible causes of poor immune responses could not identify one single explanation and suggested multifactorial causes, including immunosuppression by anti-malarial treatments such as chloroquine and sulfadoxine/pyrimethamine [77].

In 1996, the fifth case, an Israeli soldier, died a few weeks after being bitten, while asleep, by an unidentified small animal, which according to his description seemed to be a rat or a mouse [62]. No PEP was administered as transmission of rabies from rodents had never been described in Israel. Five weeks later, he complained of fever and nausea with interchanging periods of rage and calm, confusion, and water aversion, and his condition deteriorated gradually, leading to deep coma and death [66]. In 1999, another study compared the genomic sequence of a sample collected from this Israeli soldier [61]. The viral isolate was similar to virus isolates from 10 foxes, one jackal, and four cattle in the same region [61].

More recently, the death of a US soldier (the sixth reported case) in August 2011, following a bite from a stray dog in Afghanistan, sparked significant criticism of the US miliary’s handling of rabies prevention [45]. The 24-year-old Army specialist had been bitten on the hand by a feral dog in January 2011 while attempting to break up a dog fight between the stray dog and one of the local unauthorized dogs [42,45]. In August 2011, he initially experienced neck and shoulder pain, along with paraesthesias of the right arm and hand. Over the following days, he developed fever, nausea, vomiting, and dysphagia. A few days later, he was hospitalized with severe aerophobia, hydrophobia, agitation, ataxia, and syncope, and subsequently required endotracheal intubation for airway protection. Dysautonomia and fixed dilated pupils were noted, and a complete heart block required temporary pacemaker placement. A nuchal skin biopsy detected rabies virus antigens in hair follicles specimens by direct immunofluorescence, and rabies viral RNA in saliva and cerebrospinal fluid (CSF) were detected by reverse transcriptase–polymerase chain reaction (RT-PCR). The viral RNA sequence was compatible with a canine rabies virus variant associated with dogs in Afghanistan. On day 13, the patient died, after having been diagnosed with severe intracerebral haemorrhage [42]. It was reported that a rabies vaccine series had been initiated but subsequently discontinued because the vaccine had expired. However, there was no record of medical care being sought or of any animal being tested for rabies. His banked serum specimen from May 2011, tested at CDC in August 2011, did not contain VNAs, further indicating that the patient had not received PEP [49].

Among these six cases, the onset of rabies appears to be linked to:

- Failure of PEP in Case 2,
- Failure of PrEP, together with failure to recognize the risk of exposure in Case 4
- Failure to identify the risk of rabies associated with an unusual biting animal species in Case 5
- Likely failure to recognize the risk of exposure in Case 6.

Insufficient information was available to determine the contributing factors in Cases 1 and 3.

### 3.4. Potential Exposure Risks in Military Settings

For RABV, the main risk of human infection in military personnel is through the bite of an infected dog, as in civilians [1,5]. Among service members, dog bites are most commonly inflicted by personal pets or military working dogs (MWD), which may cause exposure to RABV in countries that are not free from rabies [10,60,74,76,78,79]. Cat scratches can also represent a potential rabies exposure route [43,60,72].

Less frequently, bites from other carnivores may constitute a source of potential rabies exposure. For example, Haviv et al. reported an incident in which seven Israel Defense Forces soldiers were bitten by a rabid fox [60]. A British soldier was bitten by a wild animal provisionally identified as a spotted hyena (*Crocuta Crocuta*) during training activities in Kenya and subsequently received rabies PEP [26]. Bites from nonhuman primates, particularly Rhesus macaques (*Macaca mulatta*), a species native to Afghanistan and commonly kept as a pet, have also been reported among US military personnel deployed to the region [9].

In South America, hematophagous bats are now the primary source of human exposure to lyssaviruses, as in French Guiana [1,21]. French troops conducting deep-forest operations in the Amazon rainforest are occasionally exposed to nocturnal bites to the toes during sleep, despite recommendations to keep the feet inside bed nets (unpublished data from French military veterinarians). Similarly, bat exposures occurring during sleep were reported among US Airmen in the United States, where dog-mediated rabies had been eliminated, although other RABV variants continued to circulate in wildlife [63].

During deployment, military duties frequently require extensive outdoor activities, which increase the likelihood of contact with stray dogs or wild animals that may transmit rabies [7,8,9,10,11,59]. This risk is further amplified by the “mascot phenomenon” [9,10,45,48,64]. Under stressful conditions, such as isolation from family and friends, lack of comfort, excess free time, service members may be drawn to local fauna, either out of curiosity for exotic animals such as monkeys or certain reptiles, or from a psychological need for affective contact with familiar pets, such as cats or dogs [48]. These mascots represent a risk during deployment and sometimes also post deployment, if exported to non-endemic countries.

### 3.5. Preventing Exposure

As noted in the previous paragraph, military personnel are exposed to rabies because of contact with various animal categories: MWD, mascots, feral animals and wild animals. Measures to prevent human exposure depend on these categories. MWD are not the most exposed. Indeed, as suggested by NATO [80], specified by European legislation [81] and reported in several articles, the US, European and other Armed Forces routinely vaccinate their dogs, at least when being deployed to areas where rabies is endemic [8,57,69]. For MWD deployed from Europe, regulations require verification of vaccine efficacy by measuring neutralizing antibody levels. Apart from European regulation, only one study in South Korea investigated the vaccination of military dogs. Kim et al. [69] found RABV antibody titers above the protective threshold in 97.4% of tested dogs (*n* = 78). None of the selected publications addressed the management of MWD bitten by animals suspected of having rabies. The dogs should receive a rabies booster vaccination and undergo veterinary observation, with regular follow-up examinations after the bite.

Numerous selected publications mentioned stray animals and wildlife as sources of rabies exposure [45,50,51,63,64,72]. Stray dog and cat populations may increase substantially in countries affected by armed conflict. These animal reservoirs were responsible for most human rabies cases and were a frequent source of exposure requiring PEP among military personnel [7,75]. Several measures were described in the literature to reduce contacts between military personnel and stray animals and wildlife, including bats. In the US Army, feral and wild animals were trapped and euthanised on US bases both domesticaly and abroad [46,51]. In the Iraq theater of operations, 14 000 dogs and cats were euthanised in 2005 [51]. Additional measures may be implemented depending on the base environment, including reinforcing perimeter fences and walls and restricting animal access to food sources through proper waste management [45,50,51,63,64,72]. Specific measures were adopted at a US base in Texas to prevent a US Air Force basic training squadron from potential exposures to rabies virus carried by bats (*Tadarida brasiliensis*) in sleeping areas. A colony of these bats (estimated at 400–600) had been nesting inside wall crevices of the building for several years and had recently entered the sleeping bays. Renovation work on the building, in collaboration with a specialist, successfully prevented bats from entering without the need to capture them [63]. In French Guiana, military recommendations for preventing bites from desmodine bats include closing openings in huts, installing screens over windows, properly securing hammock mosquito nets and keeping the feet inside the net while sleeping, increasing the thickness of protective clothing (e.g., socks and poncho liners), wearing white socks to facilitate the detection of blood in the morning in case of a nocturnal bite, deploying bat-deterrent devices around the perimeter of the camp (e.g., red lights or candles and sheets of lightly crumpled aluminum foil suspended from tree branches), and avoiding caves inhabited by bat colonies [82]. In Dayr Kifa, Lebanon, French peacekeepers serving with the United Nations (UN) observed a significant increase in stray cats, resulting in a potentially higher risk of rabies exposures due to cat scratches [72]. The strategy to reduce the feline population in accordance with UN policies of animals welfare was to trap, sterilize and relocate them to a shelter more than 40 km away. However, the effectiveness of these measures was not assessed in the published literature.

During the initial stages of military deployment, strict orders are given to avoid any contact with wild and stray animals [45,51]. In the French Armed Forces several texts have been written since 2009 to regulate this practice [82]. This ban also applies in the US military [45]. However, as each deployment progresses, these restrictions become less stringent and less strictly enforced [45,50], resulting in a population of mascots that pose a major rabies risk. Indeed, the health status of these mascots remains unclear, particularly on international military bases [10,64], where they may simply be tolerated [48,64] or cared for by military veterinary services [45,79]. At a UN camp in Egypt in 2016, the coexistence of authorized, vaccinated mascots and unofficial mascots led to the misperception that interaction with any animal on the base was permitted and safe [10]. Mascots are sometimes tolerated because they can help manage stress for deployed troops or serve as sentinels [48]. However, when they are unofficial and not medically supervised, they may pose a risk of rabies exposure, potentially resulting in tragic consequences. In 2014, an informal mascot at the international camp of Warehouse, Afghanistan, bit three soldiers before dying of rabies, resulting in 412 exposed individuals [64]. In 2008, 26 dogs and cats from Iraq were shipped to USA to be reunited with the personnel who had befriended them in the theater [45]. After arrival, one of the dogs died of rabies after exposing both humans and other animals. None of the 24 dogs had the required valid rabies vaccination certificates and the vaccinal status of the two cats was not reported. As the presence of mascots cannot be avoided during long-term deployments, giving them an official status may be a strategy to reduce the risks. They must undergo regular health monitoring by a military veterinarian, receive adequate prophylaxis and be visually recognizable (specific collar for example) to avoid confusion with an unauthorized animal. Adherence to strict safety measures is a key first-line prevention strategy.

### 3.6. Human Pre-Exposure Prophylaxis (PrEP)

Because exposure to potentially rabid animals cannot always be avoided, some military forces recommend rabies vaccination as PrEP. Although NATO has recommended rabies vaccination for selected military categories at increased risk (e.g., veterinarians, MWD handlers) [83] and some countries have implemented this recommendation [7,49,72], rabies preparedness in military settings remains shaped by country-specific policies, available resources and epidemiological conditions. Armed forces must therefore balance these considerations. On the one hand, PrEP greatly simplifies PEP, by eliminating the need for RIG and reducing the number of rabies PEP vaccine doses [11,49]. On the other hand, systematic vaccination of all deployed soldiers may represent a substantial financial burden.

In Belgian, Dutch, Finnish, German and Polish armed forces, rabies PrEP is mandatory before deployment to endemic regions [49,65,71,72]. In France, United-Kingdom, South Korea, and USA, PrEP is mandatory for personnel at high risk of exposure (e.g., veterinarians and MWD handlers) [53,59,70,72].

To reduce costs and improve compliance, several studies have been conducted in military personnel (9/42, 21%) providing valuable study cohorts. These studies have evaluated the efficacy of PrEP using ID administration, which requires lower doses of vaccine than the intramuscular route [56,65,76]. Since 2009, the Belgian Defense has routinely used ID rabies PrEP, initialy with three single ID doses on days 0, 7, and 21 or 28, and since 2018, the Belgian and Dutch Defense have implemented a PrEP regimen consisting of two double ID doses on days 0 and 7 [49]. Several studies have evaluated the efficacy of these two protocols, using varying intervals between inoculations and combinations of ID and IM administration, including booster doses administered after one year or more [66,67,68]. Seroconversion rates, assessed from the day of the second vaccine administration to more than one year thereafter for the regimen involving two ID injections at each visit, and from day 2 to day 365 after the last vaccine dose for the three-dose ID regimen, ranged from 98.6% to 100%. For both ID regimens, immune responses were stronger when vaccine doses were administered later than originally scheduled [67,68]. Since 2018, WHO has recommended two double-dose ID or two single-dose IM regimens, on days 0 and 7. It considers that the immunological priming induced by a two-dose pre-exposure prophylaxis (PrEP) schedule confers long-lasting, potentially lifelong immunity, and therefore does not recommend additional booster doses, although national guidelines may differ [65,68].

Routine measurement of antibodies following rabies PrEP is not recommended by the WHO in service members, although serologic testing is mandatory after ID regimens in Canada and Australia [49].

### 3.7. Post-Exposure Prophylaxis (PEP)

In the military literature, PEP was the most challenging aspect of rabies management in troops. Among the selected publications on PEP (8 of 14 papers from the US, UK, French, and South Korean armed forces cf Table 4), military medical services may use the WHO exposure classification to guide management. However, difficulties remain in accurately assessing rabies exposure risk. The decision to initiate PEP was based on prior reporting of a bite or scratch to healthcare workers. All selected publications on PEP in armed forces suggested that bite incidents were underreported, both during deployment and in other settings. Indeed, minor bite or scratch injuries classified as category II, which represented the majority of cases, often went unreported or were reported late, as affected service members may not seek medical attention [7,8,46,53,59]. Perception of rabies risks in US military in Afghanistan increased following the death of a soldier from rabies [9]. Over a four-month period, 126 bites were reported [9], suggesting that the 643 animal bites reported among all deployed US service members between 2001 and 2010 substantially underestimated the true burden of animal bites [7]. This is further supported by 899 reported bites between 2011 and 2018 [8].

Even when the risk was acknowledged, timely access to rabies PEP could be limited or delayed under field conditions [43,46]. Potential rabies exposures may interrupt operational duties and necessitate medical evacuation, thereby affecting mission readiness and requiring additional medical resources [43,46]. Administration of RIG appeared to be the most challenging aspect of PEP implementation, with reports of RIG being unavailable or administered more than 10 days after exposure [53,75]. Management of unplanned vaccinations and insufficient RIG stocks rapidly became a major challenge, while ongoing conflict further hampered logistics. The decision to initiate rabies PEP involves a complex, multifactorial risk assessment, with potentially fatal consequences if the risk is underestimated [59,72]. On the other hand, it is important to minimize the use of vaccine and RIG in order to preserve these critical resources. However, no ready-to-use protocol is available, particularly in newly established theaters of operation, where medical workers may not have received predeployment training in rabies management.

The lack of robust animal rabies surveillance in conflict-affected countries complicates the assessment of rabies risk following an exposure, especially when unusual animal species are involved. Rabies incidence may increase when animal health control measures and surveillance can no longer be implemented, as observed in Ukraine [12].

Moreover, the status of the animal responsible for a potential human exposure is more frequently unknown during international deployments than in non-deployment settings [7,8]. In very rare cases, dogs and cats may be observed after exposure during deployment, according to the regulations of each country, for up to 10 days (USA) or 15 days (France) to monitor for signs of rabies when feasible. For unwanted or stray animals, euthanasia followed by brain testing may be considered to discontinue PEP if the test result is negative [10]. Rabies diagnosis using the gold standard FAT is challenging during deployment. Consequently, before deployments to Iraq and Afghanistan, US military veterinary services established a partnership with a laboratory in Germany to perform rabies diagnostic testing and deployed a direct rapid immunohistochemical test in the field (DRIT); however, this test could not be used for cases involving human exposure [52]. The severed animal heads were also sent to a US military laboratory in San Francisco for diagnostic testing on behalf of the US naval base at Subic Bay in the Philippines [58].

Finally, to support the decision-making process, rabies boards were established in several theaters of operation, including Clark Air Base in the Philippines [50] and other US air bases [61]. These boards consisted of a physician and a veterinarian who assessed the risk of rabies following an animal exposure and jointly decided on PEP initiation and the management of the culprit when captured. Multidisciplinary technical collaboration among specialists from different fields was recommended for the management of rabies risks globally, particularly in newly established operational theaters [46].

## 4. Discussion

Our study identified only six reported cases of rabies in the armed forces between 1952 and February 2026, suggesting either effective preventive measures or case underreporting. Although restricting our search to published scientific literature may have limited the number of detected cases, this approach enhanced the reliability and robustness of the data, particularly because distinguishing accurate information from misinformation is increasingly challenging for sensitive topics such as military operations. This limitation is shared by other reviews of military medical topics [84]. The US armed forces were overrepresented in our study, likely due to their large number of service members and the predominance of scientific publications in English. Military-focused publications peaked during periods of war and international deployment [85]. Some nations may also have deliberately refrained from publishing military health data to protect operational security, thereby limiting data availability and reducing transparency.

Regarding rabies in military settings, published reports of human cases originated from countries with no or few reported human cases [44,54,55,61,62,73,76]. When rabies infections occurred during or in connection with international deployment, they were sufficiently rare and noteworthy to warrant publication. In contrast, in the armed forces of rabies-endemic countries in Africa and Asia, cases occurring in military contexts may go unreported, as they often do in the civilian population. Therefore, the six rabies cases identified in military settings likely represented only a small fraction of the true burden. The extent of underreporting likely varied across nations and armed forces. As a result, the low number of reported cases may underestimate the risk perceived by deployed personnel, military medical services, and military command. The perception of rabies risk among Cases 3 and 6 was low. Neither avoided contact with stray dogs [45,55]. Case 3 did not seek medical advice for the injury caused by the puppy, unlike a second soldier bitten by the same dog, who sought medical care, received PEP and survived [55]. Case 6 intervened in a fight between two stray dogs. In Afghanistan, US Army General Order 1B specifically prohibited contact with local pets or wildlife, but this rule was not always observed [46]. Mascots were no exception [9,43,45,46] and were considered helpful for coping with the stress of deployment [86,87]. This underestimation of rabies risk among soldiers was also reflected in the underreporting of bites in endemic areas, particularly when the injury appeared minor and benign [8].

Military medical services encountered difficulties in estimating the extent of the risk during deployments due to the lack of precise data on rabies incidence in animal populations [46,59,72]. Moreover, the decision to administer PEP and determine the appropriate regimen may be challenging when the culprit animal was unknown, as illustrated by Case 5.

This decision-making process was further complicated by the need for physicians to carefully manage limited stocks of rabies vaccines and RIG, which were scarce and difficult to procure [72]. In the US military, RIG was stocked at Level II aid stations in Afghanistan, whereas vaccines were available at Level I aid stations (battalion level). Soldiers suspected of having been exposed to lyssaviruses therefore required medical evacuation [46].

**Figure 2:**
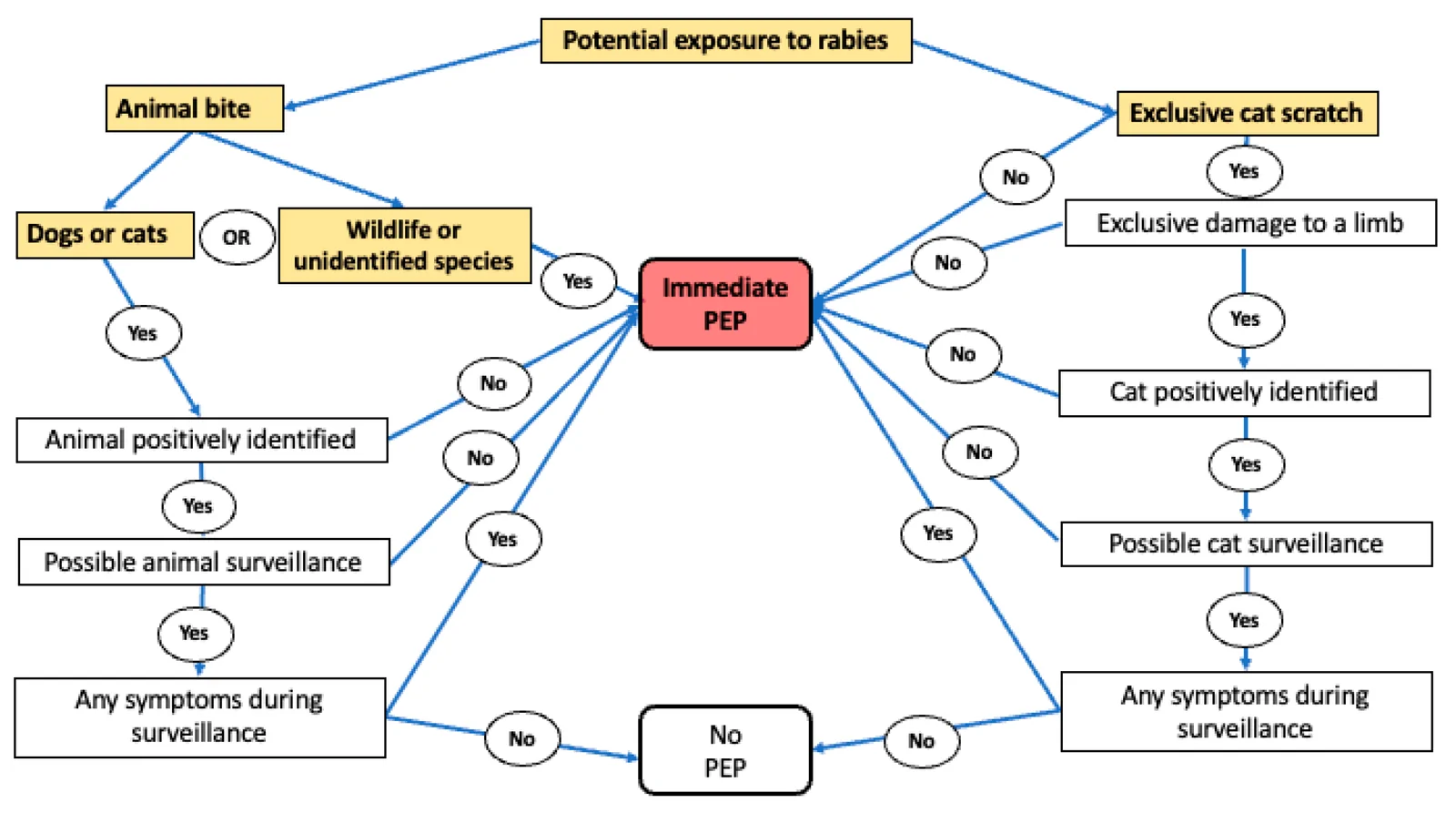
Decision-support framework for initiating PEP following Category II exposures during deployment in rabies-endemic areas, according to the type of animal exposure (adapted and extended from Gasc et al. [72]).

**Figure 3:**
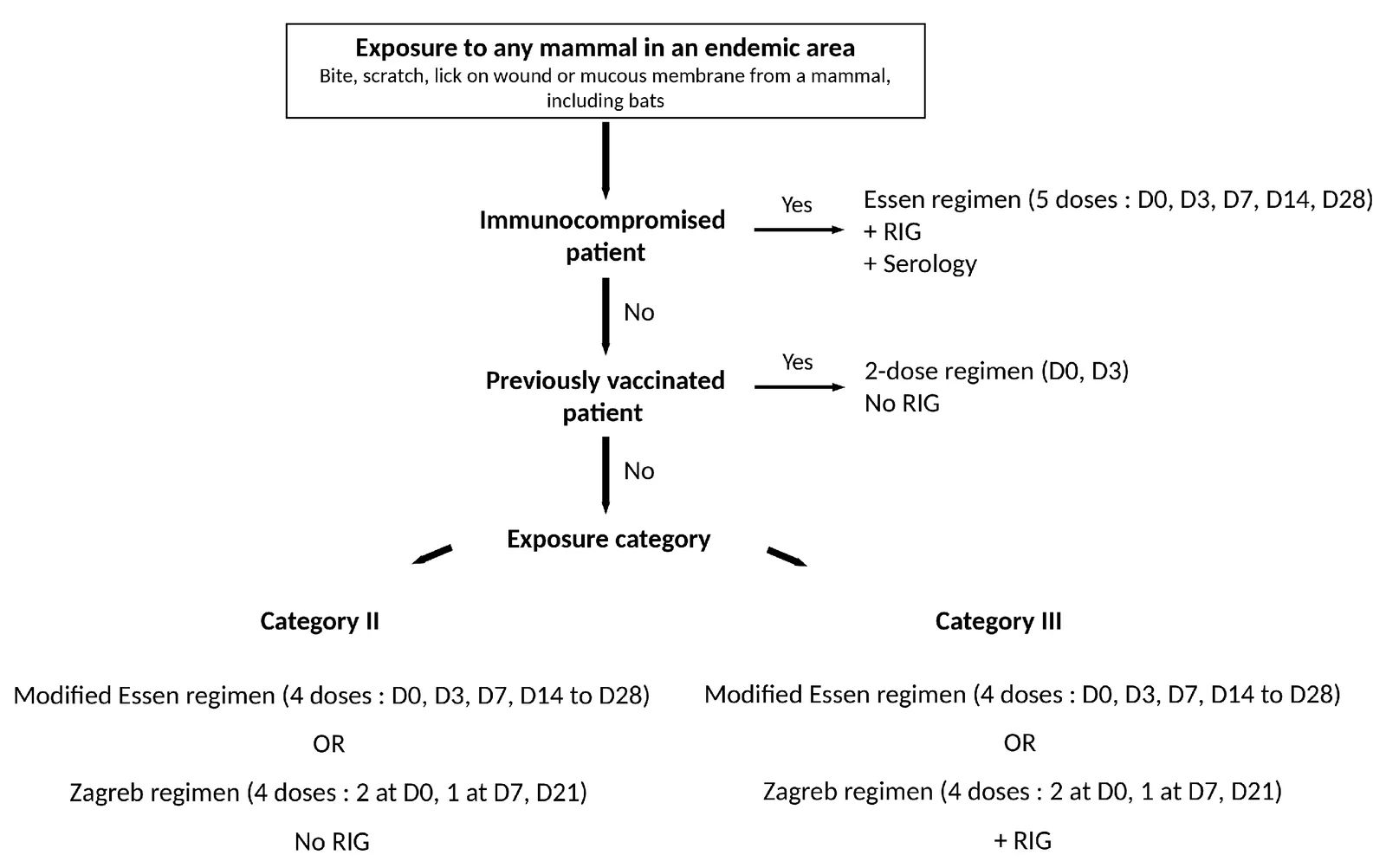
Decision-support framework for selection of rabies PEP regimen based on immune status, prior vaccination, and exposure category, including indications for rabies immunoglobulin (RIG).

Given the complexity of these decisions and the operational constraints in deployment settings, we proposed two decision-support frameworks to guide clinicians in determining whether to initiate rabies PEP and selecting the appropriate regimen (Figure 2 and Figure 3).

Figure 2 provides guidance for particularly challenging exposure scenarios in which PEP medications may need to be conserved in the field while considering the risk of rabies transmission, specifically Category II bites from dogs and scratches from cats, which may contaminate their claws through licking. Figure 3 summarizes the indications for PEP and the WHO-recommended regimens.

Beyond the decision-making challenges outlined above, the six cases also illustrated concrete difficulties and potential errors in rabies PEP management in the field. In Case 6, the administration of an expired vaccine was suggested, although this was disputed [49]. In Case 2, Dehner et al. [48] attributed the treatment failure to delayed RIG administration and possible interference with the vaccine administered concomitantly. Regarding Case 4, several hypotheses were proposed to explain the absence of detectable antibody titres eight months after completing PrEP, but it remained unclear why neither PEP nor partial PEP was administered following the puppy bite [45,60].

Finally, whether the likelihood and consequences of rabies justify the financial and logistical costs and resource requirements of providing PrEP to all deployed personnel remains a decision for the military commands of individual nations. The strategy of not systematically vaccinating deployed soldiers was questioned in the study by Moe et al. [43]. The arguments advanced were limited RIG supplies, restricted availability and the recommendations of the Advisory Committee on Immunization Practices (ACIP) [88]: “Some international travellers might be candidates for pre-exposure vaccination if they are likely to come in contact with animals in areas where dog or other animal rabies is enzootic and immediate access to appropriate medical care, including rabies vaccine and immune globulin, might be limited”. These constraints remain relevant for many deployed soldiers. The author concluded in [43] that research on ID PrEP was the next step toward enabling systematic vaccination of US soldiers before deployment. Since then, multiple ID schedules using lower doses and shorter regimens have been recommended by the WHO [5].

In the USA, rabies vaccination (military and civilian) is currently administered IM [53]. Indeed, of the 22 729 bites reported in the US Armed Forces between 2011 and 2018, only 899 (4%) were deployment-related; among these, 316 (35.2%) received rabies vaccine and 139 (15.5%) required RIG [8]. However, even with the reduced vaccine doses permitted by the ID route, reducing the cost of PrEP to a level comparable to that of PEP in large armed forces such as the US military, which operates across multiple theatres worldwide, is likely to remain challenging.

In smaller armed forces in non-endemic countries, a targeted PrEP strategy for personnel likely to be deployed to endemic areas, and therefore at risk of rabies exposure, may be more appropriate and potentially cost-effective. This approach is particularly relevant given logistical constraints, such as limited access to RIG, delayed evacuation, or remote deployments, which can make timely administration of PEP challenging after exposure.

## 5. Conclusions

Rabies, particularly dog-mediated rabies, remains a deadly threat to military personnel deployed in endemic areas. This review highlighted the challenges of rabies prevention and post-exposure management in military settings and proposed practical decision-making frameworks to assist medical personnel in assessing exposure risk, determining when PEP is indicated, and selecting the appropriate PEP regimen. The recent introduction of shorter, low-dose ID PrEP schedules recommended by the WHO has the potential to substantially reduce the financial, logistical, and operational constraints associated with PrEP, thereby improving its feasibility for military personnel at risk of rabies exposure. Regardless of the command policy on PrEP, raising awareness of rabies risk among soldiers, reinforcing education on preventive measures, and ensuring timely access to PEP remain essential. Ultimately, every effort should be directed toward preventing human rabies cases, as no deaths from this vaccine-preventable disease are acceptable when safe and effective prophylactic measures are available.

## Figures and Tables

**Table 1:** Main laboratory tests used in rabies diagnosis.

	Test	Sensitivity (Se)/Specificity (Sp)	Samples	Species	Post (P) Ante (A) Mortem	Technical Comments	Ref.
Antigens	DFA	High; gold standard	Brain	All	P	Fluorescence microscopy	[23,39]
		Less Se than in brain	Skin, corneal smear	Human	A		[40]
	DRIT		Brain	Animals	P	None	[38]
	Lateral flow assay	Varied Se and Sp, lack of standardization	Brain	All	P	None	[39]
	Negri bodies by histology	Low Se	Brain	All	P	None	[39]
Antibodies	FAVN, RFFIT	Antibody production occurs later in disease progression, low Se, poor sera quality decrease FAVN Sp	CSF, serum	Human	A, not suitable for diagnosing rabies	Cell culture, live virus in biosafety containment, fluorescence microscope	[38,39]
	ELISA					Not validated for international animal movement or trade	[39]
Nucleic acid	PCR, RT-PCR	High	Brain	All	P	None	[31]
		Lower Se and Sp than in brain postmortem	CSF, Saliva, skin	Human	A	None	[39]
Infectious virus	RTCIT	Low sensitivity	Brain	All	P	Biosafety containment facilities	[39]
	MIT	Acceptable	Brain	All	P	Biosafety containment facilities and ethically challenging	[38]

DFA: Direct fluorescent antibody test; DRIT: direct rapid immunohistochemical test; FAVN: fluorescent antibody virus neutralisation test; RFFIT: rapid fluorescent focus inhibition test; ELISA: enzyme-linked immunosorbent assay; PCR: polymerase chain reaction; RT-PCR: reverse transcription polymerase chain reaction; RTCIT: rabies tissue culture infection test; MIT: mouse inoculation test; CSF: cerebrospinal fluid; Se: sensitivity; Sp: specificity; A: antemortem; P: postmortem.

**Table 2:** World Health Organization rabies post-exposure recommendations for unvaccinated people in regions with endemic rabies [5].

Category of Exposure to Suspect Rabid Animal	Recommended PEP
Category I (no exposure) Touching or feeding animals, licks on intact skin	None
Category II (exposure) Nibbling of uncovered skin, minor scratches or abrasions without bleeding	Immediate vaccination and local treatment of wound
Category III (severe exposure) Single or multiple transdermal bites or scratches, contamination of mucous membrane or broken skin with saliva from animal licks, exposures because of direct contact with bats	Immediate vaccination, administration of RIG and local treatment of wound

**Table 3:** Inclusion and exclusion criteria to identify articles suitable for full review.

Inclusion Criteria	Exclusion Criteria
Original studies	Editorials
Case reports	Letters to the editor
Surveillance and epidemiology reports	Correspondences
Review articles	Commentaries
Addressing rabies, rabies risk or animal bites incidents among military populations and US Peace Corps	Focused solely on veterinary or non-human studies
	Did not involve military populations or military contexts
	Articles not available in full text

**Table 4:** Characteristics of included studies (*n* = 42).

		Number of Articles	References
Type of literature	Official reports	2	[42,73]
	Military publications	16	[7,8,10,26,43,44,45,46,47,48,49,50,51,52,53,75]
	Scientific publications	24	[9,11,12,54,55,56,57,58,59,60,61,62,63,64,65,66,67,68,69,70,71,72,74,76]
Country	Belgium	3	[49,67,68]
	France	3	[64,70,72]
	Israel	3	[60,61,62]
	South Korea	2	[53,69]
	Netherlands	3	[54,65,66]
	Poland	1	[71]
	UK	2	[26,59]
	Ukraine	1	[12]
	USA	22	[7,8,9,10,42,43,44,45,46,47,48,51,52,55,56,57,58,63,73,74,75,76]
	Several	2	[11,50]
Area	Domestic deployment	8	[12,57,60,61,62,63,69,74]
	Overseas deployment	19	[9,10,26,42,43,44,46,48,50,51,52,54,55,58,59,64,72,73,75]
	Worldwide	6	[7,8,11,45,47,76]
	Not applicable	9	[49,53,56,65,66,67,68,70,71]
Subject	Human cases	9	[26,42,43,44,54,55,60,61,62]
	Vaccine protocol trial	9	[49,56,65,66,67,68,70,71,76]
	Epidemiological studies on PEP	14	[7,8,9,10,12,46,50,53,58,59,63,72,74,75]
	Animal rabies management	5	[51,52,64,69,73]
	Larger reviews including rabies	5	[11,45,47,48,57]

UK: United Kingdom; USA: United States of America; PEP: post-exposure prophylaxis.

**Table 5:** Reported rabies cases in military settings.

	Case 1	Case 2	Case 3	Case 4	Case 5	Case 6
Year	1950	1967	1973	1983	1996	2011
Nationality	Dutch	American	American	American	Israeli	American
Sex, age	M, 27	M, 20	M, NS	F, 23	M, 20	M, 24
Country of exposure	Indonesia	Vietnam	Vietnam	Kenya	Israel	Afghanistan
Animal	NS	Dog	Dog	Dog	Rodent?	Dog
PrEP	NS	None	None	3 ID vaccine	None	NS
PEP	NS	RIG and vaccine	None	None	None	None
Ref	[54]	[44]	[55]	[76]	[61,62]	[46,49]

NS: Not specified.

## Data Availability

No new data were created or analyzed in this study. Data sharing is not applicable to this article.

## References

[1] WHO Fact-Sheet Rabies. https://www.who.int/news-room/fact-sheets/detail/rabies.

[2] Embregts C.W.E., Begeman L., Voesenek C.J., Martina B.E.E., Koopmans M.P.G., Kuiken T., GeurtsvanKessel C.H. (2021). Street RABV Induces the Cholinergic Anti-Inflammatory Pathway in Human Monocyte-Derived Macrophages by Binding to nAChr A7. Front. Immunol..

[3] Hemachudha T., Laothamatas J., Rupprecht C.E. (2002). Human Rabies: A Disease of Complex Neuropathogenetic Mechanisms and Diagnostic Challenges. Lancet Neurol..

[4] John B., Kumar S., Kumar S., Dalal S.S., Mohimen A. (2020). Child Survivor of Rabies in India: A Case Report. Paediatr. Int. Child Health.

[5] WHO Rabies Vaccines: WHO Position Paper 2018. https://www.who.int/publications/i/item/who-wer9316.

[6] Gautret P., Parola P. (2012). Rabies Vaccination for International Travelers. Vaccine.

[7] (2011). Armed Forces Health Surveillance Center Animal Bites, Active and Reserve Components, U.S. Armed Forces, 2001–2010. MSMR.

[8] Williams V.F., Taubman S.B., Stahlman S. (2019). Animal Bites and Rabies Post-Exposure Prophylaxis, Active and Reserve Components, U.S. Armed Forces, 2011–2018. MSMR.

[9] Mease L.E., Baker K.A. (2012). Monkey Bites among US Military Members, Afghanistan, 2011. Emerg. Infect. Dis..

[10] Mease L.E., Whitman S., Lawrence R. (2018). Brief Report: Increased Number of Possible Rabies Exposures among US Healthcare Beneficiaries Treated in Military Clinics in Southern Germany in 2016. MSMR.

[11] Biselli R., Nisini R., Lista F., Autore A., Lastilla M., De Lorenzo G., Peragallo M.S., Stroffolini T., D’Amelio R. (2022). A Historical Review of Military Medical Strategies for Fighting Infectious Diseases: From Battlefields to Global Health. Biomedicines.

[12] Kannan A., Chen R., Akhtar Z., Sutton B., Quigley A., Morris M.J., MacIntyre C.R. (2024). Use of Open-Source Epidemic Intelligence for Infectious Disease Outbreaks, Ukraine, 2022. Emerg. Infect. Dis..

[13] Hurmuzache M., Gradinaru M.A., Bărbuceanu F., Moțiu R., Popescu R., Lutic A., Müller T., Freuling C.M., Vuta V. (2025). Death in the EU/EEA from Autochthonous Human Rabies, Romania, July 2025: A Call for Action. Euro Surveill. Bull. Eur. Sur Mal. Transm. Eur. Commun. Dis. Bull..

[14] Dietzgen R.G., Kuzmin I.V. (2012). Rhabdoviruses: Molecular Taxonomy, Evolution, Genomics, Ecology, Host-Vector Interactions, Cytopathology, and Control.

[15] Dean D.J., Baer G.M., Thompson W.R. (1963). Studies on the Local Treatment of Rabies-Infected Wounds. Bull. World Health Organ..

[16] McElhinney L.M., Marston D.A., Brookes S.M., Fooks A.R. (2014). Effects of Carcase Decomposition on Rabies Virus Infectivity and Detection. J. Virol. Methods.

[17] ICTV International Committee on Taxonomy of Viruses. https://ictv.global.

[18] Davis B.M., Rall G.F., Schnell M.J. (2015). Everything You Always Wanted to Know About Rabies Virus (But Were Afraid to Ask). Annu. Rev. Virol..

[19] Davis A.D., Rudd R.J., Bowen R.A. (2007). Effects of Aerosolized Rabies Virus Exposure on Bats and Mice. J. Infect. Dis..

[20] Wei Y., Li D., Yang Z., Chen K., Pan X., Xu J., Chen S. (2023). One Health Responses to Prevent the Occurrence of Rabies Due to Attacks by a Rabid Stray Dog. Vet. Med. Sci..

[21] Berger F., Desplanches N., Baillargeaux S., Joubert M., Miller M., Ribadeau-Dumas F., Spiegel A., Bourhy H. (2013). Rabies Risk: Difficulties Encountered during Management of Grouped Cases of Bat Bites in 2 Isolated Villages in French Guiana. PLoS Negl. Trop. Dis..

[22] Blanton J.D., Wallace R.M. (2015). The Ancient Curse: Rabies. Microbiol. Spectr..

[23] WHO (2018). WHO Expert Consultation on Rabies: Third Report: WHO Technical Report Series.

[24] Robardet E., Bosnjak D., Englund L., Demetriou P., Rosado Martín P., Cliquet F. (2019). Zero Endemic Cases of Wildlife Rabies (Classical Rabies Virus, RABV) in the European Union by 2020: An Achievable Goal. Trop. Med. Infect. Dis..

[25] Briggs D.J., Schweitzer K. (2001). Importation of Dogs and Cats to Rabies-Free Areas of the World. Vet. Clin. North Am. Small Anim. Pract..

[26] Ross J., Raitt S. (2012). Management of a Wild Animal Bite in a Rabies Enzootic Area. J. R. Army Med. Corps.

[27] Banyard A.C., Tordo N. (2018). Rabies Pathogenesis and Immunology: -EN- Rabies Pathogenesis and Immunology -FR- Pathogenèse et Immunologie de La Rage -ES- Patogénesis e Inmunología de La Rabia. Rev. Sci. Tech. OIE.

[28] Fisher C.R., Streicker D.G., Schnell M.J. (2018). The Spread and Evolution of Rabies Virus: Conquering New Frontiers. Nat. Rev. Microbiol..

[29] Macedo C.I., Carnieli P., Brandão P.E., Rosa E.S.T.D., Oliveira R.D.N., Castilho J.G., Medeiros R., Machado R.R., Oliveira R.C.D., Carrieri M.L. (2006). Diagnosis of Human Rabies Cases by Polymerase Chain Reaction of Neck-Skin Samples. Braz. J. Infect. Dis..

[30] Rupprecht C.E. (2025). Rabies in Animals. https://www.msdvetmanual.com/nervous-system/rabies/rabies-in-animals#epidemiology_v83306256.

[31] Jackson A. (2011). Update on Rabies. Res. Rep. Trop. Med..

[32] Ghosh J., Roy M., Lahiri K., Bala A., Roy M. (2009). Acute Flaccid Paralysis Due to Rabies. J. Pediatr. Neurosci..

[33] Robardet E., Borel C., Moinet M., Jouan D., Wasniewski M., Barrat J., Boué F., Montchâtre-Leroy E., Servat A., Gimenez O. (2017). Longitudinal Survey of Two Serotine Bat (Eptesicus Serotinus) Maternity Colonies Exposed to EBLV-1 (European Bat Lyssavirus Type 1): Assessment of Survival and Serological Status Variations Using Capture-Recapture Models. PLoS Negl. Trop. Dis..

[34] O’Shea T.J., Cryan P.M., Cunningham A.A., Fooks A.R., Hayman D.T.S., Luis A.D., Peel A.J., Plowright R.K., Wood J.L.N. (2014). Bat Flight and Zoonotic Viruses. Emerg. Infect. Dis..

[35] Amengual B., Bourhy H., López-Roig M., Serra-Cobo J. (2007). Temporal Dynamics of European Bat Lyssavirus Type 1 and Survival of Myotis Myotis Bats in Natural Colonies. PLoS ONE.

[36] Johnson N., Aréchiga-Ceballos N., Aguilar-Setien A. (2014). Vampire Bat Rabies: Ecology, Epidemiology and Control. Viruses.

[37] George D.B., Webb C.T., Farnsworth M.L., O’Shea T.J., Bowen R.A., Smith D.L., Stanley T.R., Ellison L.E., Rupprecht C.E. (2011). Host and Viral Ecology Determine Bat Rabies Seasonality and Maintenance. Proc. Natl. Acad. Sci. USA.

[38] WHO (2018). Laboratory Techniques in Rabies.

[39] World Organisation for Animal Health (WOAH) (2024). Manual of Diagnostic Tests and Vaccines for Terrestrial Animals, Chapter 3.1.19. Rabies.

[40] Warrell M.J., Looareesuwan S., Manatsathit S., White N.J., Phuapradit P., Vejjajiva A., Hoke C.H., Burke D.S., Warrell D.A. (1988). Rapid Diagnosis of Rabies and Post-Vaccinal Encephalitides. Clin. Exp. Immunol..

[41] Embregts C.W.E., Salahuddin N., Gohar M.A., Naseer F., Hussain S., Alblas K., Voermans J.J.C., Guldemeester J., Oude Munnink B.B., Aijaz J. (2026). Detection of Rabies Virus RNA in Dog-Bite Wounds in a Rabies-Endemic Area: Evidence from an Observational Cohort Study. eBioMedicine.

[42] (2012). Centers for Disease Control and Prevention (CDC) Imported Human Rabies in a U.S. Army Soldier—New York, 2011. MMWR Morb. Mortal. Wkly. Rep..

[43] Moe C.D., Keiser P.B. (2014). Should U.S. Troops Routinely Get Rabies Pre-Exposure Prophylaxis?. Mil. Med..

[44] Dehner L.P. (1970). Human Rabies Encephalitis in Vietnam. Ann. Intern. Med..

[45] Cooper E.D., Debboun M. (2012). The Relevance of Rabies to Today’s Military. US Army Med. Dep. J..

[46] Dainty L.A., Morgan S.A., Parker M.E., Burke R.L. (2013). Rabies, Readiness, and Role 1 Medical Care. Mil. Med..

[47] Hoke C.H. (2005). History of U.S. Military Contributions to the Study of Viral Encephalitis. Mil. Med..

[48] Garges E.C., Taylor K.M., Pacha L.A. (2016). Select Public Health and Communicable Disease Lessons Learned During Operations Iraqi Freedom and Enduring Freedom. US Army Med. Dep. J..

[49] Van Nieuwenhove M.D.M., Damanet B., Soentjens P. (2019). Timing of Intradermal Rabies Pre-Exposure Prophylaxis Injections: Immunological Effect on Vaccination Response. Mil. Med..

[50] Rizzolo J., Osborne D.V. (1962). Current Rabies Problems of Military Medicine in SEATO Areas. Mil. Med..

[51] Dunton R.F., Sargent G.R. (2009). Addressing Feral and Wild Animal Threats during Deployment: The Distinction between Animal Control and Rabies Control. US Army Med. Dep. J..

[52] Saturday G.A., King R., Fuhrmann L. (2009). Validation and Operational Application of a Rapid Method for Rabies Antigen Detection. US Army Med. Dep. J..

[53] Park D.H., Kim S.Y., Seong G., Oh H.S. (2026). A Clinical Analysis of Rabies Post-Exposure Prophylaxis After Animal Bite Cases in the Republic of Korea Armed Forces. Mil. Med..

[54] De Vries E., Van Wermeskerken W.M. (1952). [Rabies in a soldier repatriated from Indonesia]. Ned. Tijdschr. Geneeskd..

[55] Koch F.J., Sagartz J.W., Davidson D.E., Lawhaswasdi K. (1975). Diagnosis of Human Rabies by the Cornea Test. Am. J. Clin. Pathol..

[56] Rosa F.W. (1983). Pre-Exposure Prophylaxis in Peace Corps Volunteers with Intradermal Human Diploid Cell Rabies Vaccine. J. Trop. Med. Hyg..

[57] Warner R.D. (1984). Occurrence and Impact of Zoonoses in Pet Dogs and Cats at US Air Force Bases. Am. J. Public Health.

[58] Dembert M.L., Lawrence W.B., Weinberg W.G., Granger D.D., Sanderson R.D., Garst P.D., Eighmy J.J., Wells T.E. (1985). Epidemiology of Human Rabies Post-Exposure Prophylaxis at the US Naval Facility, Subic Bay, Philippines. Am. J. Public Health.

[59] Croft A., Archer R. (1997). Dog Bites in Bosnia. Br. J. Gen. Pract. J. R. Coll. Gen. Pract..

[60] Haviv J., Rishpon S., Gdalevich M., Mimouni D., Gross E., Shpilberg O. (1999). Successful Postexposure Rabies Prophylaxis after Erroneous Starting Treatment. Prev. Med..

[61] David D., Rupprecht C.E., Smith J., Samina I., Perl S., Shram Y. (1999). Human Rabies in Israel. Emerg. Infect. Dis..

[62] Gdalevich M., Mimouni D., Ashkenazi I., Shemer J. (2000). Rabies in Israel. Public Health.

[63] Webber B.J., Ayers K.J., Winterton B.S., Yun H.C., Cropper T.L., Foster J., Kren M.C., Meek B.Y., Oliver T.A., Hudson C.M. (2014). Assessment of Rabies Exposure Risk in a Group of U.S. Air Force Basic Trainees—Texas, January 2014. MMWR Morb. Mortal. Wkly. Rep..

[64] Duron S., Ertzscheid C., De Laval F., Manet G., Ficko C., Velut G., Lefèvre F., Migliani R., Mayet A., Meynard J. (2014). Public Health Investigation in a Military Camp After Diagnosis of Rabies in a Dog—Afghanistan, 2012. J. Travel Med..

[65] De Pijper C.A., Boersma J., Terryn S., Van Gucht S., Goorhuis A., Grobusch M.P., Stijnis C. (2018). Rabies Antibody Response after Two Intradermal Pre-Exposure Prophylaxis Immunizations: An Observational Cohort Study. Travel Med. Infect. Dis..

[66] De Pijper C.A., Terryn S., Van Gucht S., Grobusch M.P., Goorhuis A., Stijnis C. (2020). Antibody Response in Dutch Marines to a Single Intramuscular Rabies Booster Immunization 1–2.5 Years after an Intradermal Pre-Exposure Schedule: An Observational Study. Travel Med. Infect. Dis..

[67] Damanet B., Costescu Strachinaru D.I., Soentjens P. (2020). Factors Influencing the Immune Response after a Double-Dose 2-Visit Pre-Exposure Rabies Intradermal Vaccination Schedule: A Retrospective Study. Travel Med. Infect. Dis..

[68] Damanet B., Costescu Strachinaru D.I., Van Nieuwenhove M., Soentjens P. (2020). Factors Influencing the Immune Response after a Single-Dose 3-Visit Pre-Exposure Rabies Intradermal Vaccination Schedule: A Retrospective Multivariate Analysis. Travel Med. Infect. Dis..

[69] Kim H.-H., Yang D.-K., Seo B.-H., Cho I.-S. (2018). Serosurvey of Rabies Virus, Canine Distemper Virus, Parvovirus, and Influenza Virus in Military Working Dogs in Korea. J. Vet. Med. Sci..

[70] Mura M., Haus-Cheymol R., Tournier J.-N. (2021). Immunization on the French Armed Forces: Impact, organization, limits and perspectives. Infect. Dis. Now.

[71] Zawadzka M., Ejchman-Pac E. (2022). Analysis of the Number and Type of Vaccinations Performed among Polish Soldiers in 2018–2021. Int. J. Environ. Res. Public. Health.

[72] Gasc T., Santinelli Y., Marti J. (2025). Pragmatic Management of Rabies Risk in Operation DAMAN 50 (Lebanon, October 2024-February 2025): A Case Study on Evidence-Based Military Field Medicine. Infect. Dis. Now.

[73] Centers for Disease Control and Prevention (CDC) (2008). Rabies in a Dog Imported from Iraq--New Jersey, June 2008. MMWR Morb. Mortal. Wkly. Rep..

[74] Hanna T.L., Selby L.A. (1981). Characteristics of the Human and Pet Populations in Animal Bite Incidents Recorded at Two Air Force Bases. Public Health Rep..

[75] Knobbe M.G., Aden B.J., Ashbaugh H.R. (2024). Comparison of Theater Medical Data Store and Reportable Medical Event Records to Theater Animal Bite Reports Submissions, 2018–2019. Mil. Med..

[76] Bernard K.W., Fishbein D.B., Miller K.D., Parker R.A., Waterman S., Sumner J.W., Reid F.L., Johnson B.K., Rollins A.J., Oster C.N. (1985). Pre-Exposure Rabies Immunization with Human Diploid Cell Vaccine: Decreased Antibody Responses in Persons Immunized in Developing Countries. Am. J. Trop. Med. Hyg..

[77] Malerczyk C., Vakil H.B., Bender W. (2013). Rabies Pre-Exposure Vaccination of Children with Purified Chick Embryo Cell Vaccine (PCECV). Hum. Vaccines Immunother..

[78] Schermann H., Eiges N., Sabag A., Kazum E., Albagli A., Salai M., Shlaifer A. (2017). Estimation of Dog-Bite Risk and Related Morbidity Among Personnel Working With Military Dogs. J. Spec. Oper. Med..

[79] Messenger R.A., Stahlman S., Chern A. (2020). Animal-Related Injuries in Veterinary Services Personnel, U.S. Army, 2001–2018. MSMR.

[80] (2018). Animal Care and Welfare and Veterinary Support During All Phases of Military Deployments.

[81] EU Regulation (EU) No 576/2013 of the European Parliament and of the Council of 12 June 2013 on the Non-Commercial Movement of Pet Animals 2013. https://eur-lex.europa.eu/eli/reg/2013/576/oj.

[82] Berger F., Watier S., Ficko C., Ponsard M.-L., Gautret P., Ringot D., Demoncheaux J.-P., Service de Santé des Armées (2018). Expositions Au Virus Rabique Dans Les Armées Françaises: Actualités et Aspects Pratiques. Médecine et Armées.

[83] NATO (2021). NATO Standards Related Document. SRD-7 to AJMedP-4. Vaccinations Catalogue within the Nato & PfP Forces, Edition A, Version 2.

[84] Bailey Z., Mahoney P., Miron M., Bricknell M. (2022). Thematic Analysis of Military Medical Ethics Publications From 2000 to 2020—A Bibliometric Approach. Mil. Med..

[85] Lile D.J., Bergman A., Rolfing J., Allard R.J., Davis T.A., Gross K.R., Elster E.A., Schwab C.W., Cannon J.W. (2024). Missing in Action: A Bibliometric Analysis of Military Research in the Medical Literature since 1950. J. Trauma Acute Care Surg..

[86] Bøg M., Filges T., Jørgensen A.M.K. (2018). Deployment of Personnel to Military Operations: Impact on Mental Health and Social Functioning. Campbell Syst. Rev..

[87] Trousselard M., Jean A., Beiger F., Marchandot F., Davoust B., Canini F. (2014). The Role of an Animal-Mascot in the Psychological Adjustment of Soldiers Exposed to Combat Stress. Psychology.

[88] Manning S.E., Rupprecht C.E., Fishbein D., Hanlon C.A., Lumlertdacha B., Guerra M., Meltzer M.I., Dhankhar P., Vaidya S.A., Jenkins S.R. (2008). Human Rabies Prevention—United States, 2008: Recommendations of the Advisory Committee on Immunization Practices. MMWR Recomm. Rep. Morb. Mortal. Wkly. Rep. Recomm. Rep..

